# Epidemiological Investigation and Antimicrobial Resistance Profiles of *Salmonella* Isolated From Breeder Chicken Hatcheries in Henan, China

**DOI:** 10.3389/fcimb.2020.00497

**Published:** 2020-09-15

**Authors:** Yaohui Xu, Xiao Zhou, Zenghai Jiang, Yaru Qi, Abdelaziz Ed-dra, Min Yue

**Affiliations:** ^1^College of Veterinary Medicine, Henan University of Animal Husbandry and Economy, Zhengzhou, China; ^2^Department of Veterinary Medicine & Institute of Preventive Veterinary Sciences, Zhejiang University College of Animal Sciences, Hangzhou, China; ^3^Zhejiang Provincial Key Laboratory of Preventive Veterinary Medicine, Hangzhou, China

**Keywords:** *Salmonella*, chicken, embryo, hatchery scale, antimicrobial resistance, prevalence, ma breed, san huang breed

## Abstract

The emergence of antimicrobial-resistant (AR) *Salmonella* has a major concern worldwide. This study was designed to determine the AR profiles and serovars distribution of *Salmonella enterica* isolated from different breeds of breeder chickens in the province of Henan, China. For this, 2,139 dead embryo samples were collected from 28 breeder chicken hatcheries, representing two domestic and four foreign breeds. The samples were subjected to the isolation and identification of *Salmonella* by PCR. The confirmed strains were serotyped according to the Kauffmann-White scheme and their AR profiles against 20 antimicrobial agents were determined by Kirby-Bauer (K-B) disc diffusion method. The results of this study showed the prevalence of *Salmonella* in 504 strains (23.56%) with a high abundance in southern regions of Yellow River (28.66%, *n* = 495, *N* = 1,727) compared to the northern regions (2.18%, *n* = 9, *N* = 412) (*p* < 0.0001). The domestic breeds were more contaminated than imported breeds (*p* < 0.0001). However, the contamination rate of samples recovered from M-hatcheries was the highest (*p* < 0.0001). Serotyping method identified 12 serovars, with the dominance of *S*. Pullorum (75.79%), followed by *S*. Enteritidis (7.14%). The AR assay showed high resistant to ciprofloxacin (77.00%), sulfisoxazole (73.00%), and ampicillin (55.60%), as well as 98.81% (*n* = 498) of the isolated strains, were resistant to at least one antimicrobial and 69.64% (*n* = 351) were resistant to three or more antimicrobials. Among them, one strain of *S*. Thompson was resistant to 15 antimicrobial agents belonging to eight different classes. In conclusion, *Salmonella* strains isolated in this study were multidrug-resistant (MDR), presenting a serious problem for human and animal health. Therefore, it is necessary to monitor, control, and rationalize the use of antimicrobials agents in chicken farms in order to limit the increasing resistance against the recent antimicrobial agents.

## Introduction

Globally, salmonellosis is considered as one of the most important zoonotic diseases. According to the estimation of the World Health Organization (WHO), non-typhoidal salmonellosis was responsible for about 1.6 billion cases of acute gastroenteritis or diarrhea, causing 3 million deaths each year (Mahmud et al., 2011). In China, a study based on the literature review estimate that the incidence of non-typhoidal salmonellosis was 626.5 cases per 100,000 persons (Mao et al., 2011; Pan et al., 2018a; Yue et al., 2020). However, many studies have reported *Salmonella* as the responsible agent of many foodborne outbreaks in China and elsewhere (Wang et al., 2007; Cleary et al., 2010; Hedican et al., 2010; Le Hello et al., 2012; Guo et al., 2015; Moffatt et al., 2016; Jourdan-Da Silva et al., 2018). Currently, animals, in particular, poultry and eggs are considered to be the primary cause for salmonellosis and numerous other foodborne outbreaks (Gieraltowski et al., 2016; Keerthirathne et al., 2017; Biswas et al., 2019, 2020; Yu et al., 2020). Generally, *Salmonella* grow in animal farms may contaminate eggs and/or meat during the slaughtering process before being transferred to humans through food chain. Indeed, previous studies have been reported the isolation of *Salmonella* from foods of animal origin as well as human samples (Ed-Dra et al., 2018; Paudyal et al., 2018, 2020; Jiang et al., 2019; Elbediwi et al., 2020). Moreover, other studies were reported the consistency relationship between *Salmonella* strains causing human diseases and those isolated at farms and/or food products (Painter et al., 2013; Pan et al., 2018b, 2019; Paudyal et al., 2019; Wang et al., 2019).

*Salmonella* are Gram-negative rod-shaped bacteria, facultatively anaerobic, and belong to the family Enterobacteriaceae. They are generally mobile, capsule-less, not spore-forming, and colonize the digestive tract of many vertebrates (Baird-Parker, 1990; Bernal-Bayard and Ramos-Morales, 2018). So far about 2,600 serovars have been discovered, many of them were implicated in human and animal diseases (Feasey et al., 2012; Gong et al., 2014; Paudyal et al., 2019). In poultry farms, *Salmonella* may contaminate the flocks causing severe diseases with a high level of mortality. *Salmonella* Gallinarum biovar Pullorum and Gallinarum are host-specific for avian species, causing Pullorum disease and fowl typhoid, resulting in huge economic losses to the poultry industries every year (Gast and Porter, 2020). However, to treat and control the bacterial infectious diseases in poultry farms, farmers use many antimicrobials for therapeutic and prophylaxis practices. Unfortunately, the antimicrobials abuse were the main driver for the emergence of multidrug-resistant (MDR) bacteria in China, which has become a serious problem to public health (Yue, 2016; Paudyal and Yue, 2019). Generally, bacteria acquired resistance in animal farms before being transferred to humans through the food chain (Mehdi et al., 2018; Elbediwi et al., 2019; Paudyal et al., 2019; Zhang et al., 2019).

China is a major consumer of chicken and its products overall the world. Henan province, located in the central part of China with convenient transportation, is also a major breeding province in China. In 2012, the export of meat, eggs, and milk from Henan province accounted for 19.14% of the country's total exports. However, foods of animal origin, in particular, contaminated poultry products (eggs and poultry meat) have been considered the main vehicles of *Salmonella* infection and were clearly associated with worldwide epidemics (Hedican et al., 2010; Guo et al., 2015; Moffatt et al., 2016). Currently, there are few studies regarding *Salmonella* of chicken origin in Henan province. Additionally, breeder farms are less studied compared to commercial farms, slaughterhouses, and markets. Therefore, in order to fill up the epidemiological gaps concerning the distribution of *Salmonella* in breeder chicken hatcheries, 28 sampling sites belonging to nine cities of Henan province were subjected to the isolation, identification, and serotyping of *Salmonella* isolates. The antimicrobial resistant (AR) patters of the isolated strains were also determined. In this study, we compared the prevalence of *Salmonella* among different hatcheries and breeds as well as investigated the prevalence of different serovars and their AR patterns for guiding the prevention and control of *Salmonella* in animals and foodborne transmission toward humans.

## Materials and Methods

### Sample Collection

From August 2014 to April 2015, a total of 2,139 dead chicken embryos were collected as the samples from randomly selected 28 hatcheries for breeding chickens in nine cities of Henan province: Zhengzhou, Xuchang, Pingdingshan, Hebi, Anyang, Zhoukou, Shangqiu, Xinyang, and Luohe, including two domestic breeds (Ma and San huang) and four foreign breeds (Hyline, Cobb, Ross 308, and Arbor Acres). According to the breeding scale, these chicken hatcheries were divided into three small-scale hatcheries (S-hatcheries, housing ≤ 10,000 chicken embryos), 22 medium-scale hatcheries (M-hatcheries, housing >10,000, ≤ 50,000 chicken embryos) and two large-scale hatcheries (L-hatcheries, housing >50,000 chicken embryos). The breeding scale of Zhoukou-3 hatchery is not available.

### Isolation

The embryo surface was disinfected with ethanol for 2 min and then placed on the sterile tray of the clean bench. Sterile forceps and scissors were used to find the yolk sac of the chicken embryo and extract the samples. The yolk sac solution was placed onto *Salmonella-Shigella* agar using sterile cotton swabs and streaked using disposable sterile inoculating loops then the plates were incubated for 24 h at 37° C. The translucent colorless or black center colonies were considered presumptive of *Salmonella* and were selected and inoculated into Luria-Bertani broth for serotyping and genomic DNA preparation (Han et al., 2011; Xin et al., 2020).

### Genomic DNA Preparation

The raw genomic DNA sample was extracted by boiling method. Briefly, 400 μL of the culture of presumptive *Salmonella* were placed in 1.5 mL tubes and centrifuged for 2 min at 12,000 rpm, then the supernatant was discarded and 200 μL of ddH_2_O were added to resuspend the culture of *Salmonella*. The suspension was centrifuged at low speed for 1 min, boiled for 10 min and transferred immediately into ice for 10 min. Then, another centrifugation was performed at 12,000 rpm for 2 min and the obtained supernatant (template DNA) was analyzed for checking the purity and stored at −20° C until use.

### PCR Identification

For the identification of *Salmonella*, amplification of enterotoxin *stn* gene was performed by PCR in a final volume of 20 μL (Xiong et al., 2018), including 10 μL of 2 × Master Mix, 1.5 μL of F/R primers (primer concentration is 5 μM, primer sequences (5′-3′) were *stn* F: TATTTTGCACCACAGCCAGC and *stn* R: CGACCGCGTTATCATCACTG), 2 μL of template DNA, and 5 μL of ddH_2_O. The PCR amplification was performed under the following reaction conditions: initial denaturation at 94° C for 5 min; 35 sequential cycles of denaturation at 94° C for 30 s, annealing at 58° C for 30 s, and extension at 72° C for 20 s; and a final extension at 72° C for 5 min. The PCR products were subjected to gel electrophoresis, and those presented a targeted band of about 260 bp were considered positives.

### Serotyping

*Salmonella* isolates confirmed by PCR were serotyped by slide agglutination test with O and H antigens (Tianrun Bio-Pharmaceutical, Ningbo, China), according to the manufacturer's instructions. The results were analyzed and interpreted according to the Kauffmann-White scheme. Molecular identification and discrimination of *S*. Pullorum and *S*. Gallinarum was performed as recommended previously (Zhu et al., 2015).

### Antimicrobial Susceptibility Test

Antimicrobial susceptibility profiles of the isolated *Salmonella* strains were determined according to the Kirby-Bauer (K-B) disc diffusion method and the recommendations of the American Clinical Laboratory Standards Institute (CLSI, 2015). For control strains, *Escherichia* coli ATCC 25922 and *Pseudomonas aeruginosa* ATCC 27853 were used. Twenty different antimicrobials belonging to 12 classes were used for this assay. The isolates showing a decrease in susceptibility (intermediate) were ranged with the resistant group for the sake of clarity and to facilitate analysis. The classes of antimicrobials used in our assay were as follow: aminoglycosides (kanamycin: KAN 30 μg, gentamicin: GEN 10 μg, amikacin: AMK 30 μg), penicillin (ampicillin: AMP 10 μg), beta-lactam combination (amoxicillin-clavulanic acid: AMC 20/10 μg), cephems (ceftriaxone: CRO 30 μg, ceftazidime: CAZ 30 μg, cefazolin: CFZ 30 μg), carbapenems (meropenem: MEM 10 μg, imipenem: IPM 10 μg), monobactams (aztreonam: ATM 30 μg), tetracyclines (tetracycline: TET 30 μg, oxytetracycline: OTC 30 μg), polypeptide (colistin: CST 10 μg), phenicol (chloramphenicol: CHL 30 μg), quinolones (enrofloxacin: ENR 5 μg, ciprofloxacin: CIP 5 μg), sulphonamides (sulfamethoxazole-trimethoprim: SXT 23.75/1.25 μg, sulfisoxazole: SIZ 250 μg), nitrofurans (nitrofurantoin: NIT 300 μg).

### Statistical Analysis

SPSS 26.0 software was used to perform statistical analysis on the prevalence of *Salmonella* in different regions, hatcheries, breeds, and serovars and biovars by using Chi-square test. The two-way ordinary ANOVA analysis was used to compare the difference in the cumulative prevalence of *Salmonella* recovered from L-hatcheries and M-hatcheries with respect to the individual antimicrobial.

## Results

### Prevalence, Breed, and Serovar Distribution

**Figure 1:**
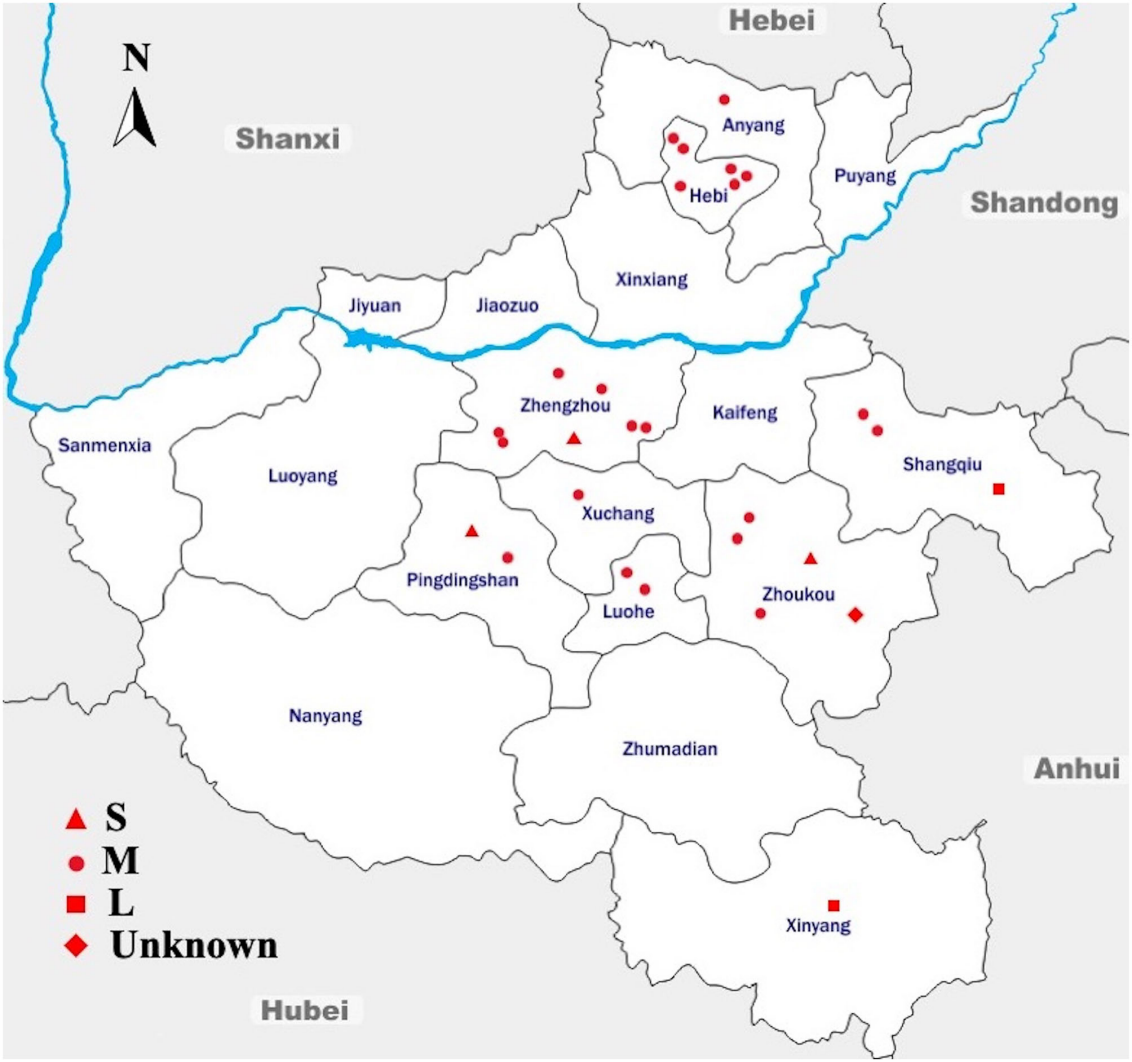
Geographic Distribution of the Sampling Sites Across Different Regions in Henan, China. Sampling sites are denoted as small-scale hatcheries (S), medium-scale hatcheries (M), large-scale hatcheries (L), and unknown-scale hatcheries (Unknown). The blue line denotes the Yellow River, flowing through the province to the east into the Bohai Sea. The arrow shows the geographical north. This map is not drawn to scale.

During the 9 months of sampling, from August 2014 to April 2015, a total of 2,139 dead chicken embryo samples were collected from 28 breeder chicken hatcheries and analyzed for the presence of *Salmonella*. The sampling framework was designed to cover a large geographical area of Henan province; in fact, 28 sampling sites belonging to nine cities were selected for this study (Figure 1).

The results of this study showed that the average prevalence of *Salmonella* was 23.56% (*n* = 504, *N* = 2,139) (95% CI: 21.8–25.4). Among the total samples collected in this study (*N* = 2,139), 142 were from S-hatcheries, 1,759 were from M-hatcheries and 180 were from L-hatcheries, in addition to 58 samples collected from an unknown-scale hatchery. The results showed that the prevalence of *Salmonella* in M-hatcheries and L-hatcheries were 27.52% [95% CI: 25.4–29.7 (*n* = 484, *N* = 1,759)] and 11.11% [95% CI: 6.9–16.6 (*n* = 20, *N* = 180)], respectively. Whereas, *Salmonella* was not detected in S-hatcheries (*n* = 0, *N* = 142) and the unknown-scale hatchery (*n* = 0, *N* = 58) (Table 1). The difference in the prevalence of *Salmonella* between different hatcheries was studied by using Chi-square test which revealed that the higher prevalence in M-hatcheries was statistically significant (*p* < 0.0001). Moreover, our results showed that the prevalence of *Salmonella* in the south of the Yellow River (28.66%, *n* = 495, *N* = 1,727) was much higher than the north region (2.18%, *n* = 9, *N* = 412) (*p* < 0.0001).

**Table 1:** The distribution of twelve serovars at different hatcheries.

**Hatcheries**	**Total samples**	**Serovars**	**Positive samples**	**%Prev. (HL)^a^**	**%Prev. (Ov.)^b^**
Scale unknown	58 (1)	–	0 (0)	**0.00**	**0.00**
S-hatcheries^c^	142 (3)	–	0 (0)	**0.00**	**0.00**
M-hatcheries^d^		Entebbe	1	0.06	0.05
		Edinburg	17	0.97	0.79
		Thompson	14	0.80	0.65
		Tennessee	9	0.51	0.42
		Tamilnadu	1	0.06	0.05
		Fillmore	1	0.06	0.05
		Gatuni	1	0.06	0.05
		Pullorum	365	20.75	17.86
		Enteritidis	36	2.05	1.68
		Blegdam	17	0.97	0.84
		Kimpese	4	0.23	0.19
		Cerro	18	1.02	0.84
Subtotal M-hatcheries	1759 (22)		484 (17)	27.52^****^	
L-hatcheries^e^		Pullorum	17	9.44	
		Gallinarum	2	1.11	0.09
		Blegdam	1	0.56	
Subtotal L-hatcheries	180 (2)		20 (1)	**11.11**	
**Total hatcheries**	**2139 (28)**		**504 (18)**		**23.56**

a *Prevalence at hatchery level*;

b *Overall prevalence*;

c *Small-scale hatcheries*;

d *Medium-scale hatcheries*;

e *Large-scale hatcheries*.

**** *p < 0.0001, statistical difference in Salmonella prevalence between M- and L-hatcheries*.

Six different breeds were studied, including two domestic breeds (Ma and San huang) and four imported breeds (Hyline, Cobb, Ross 308, and Arbor Acres). The distribution of these breeds at different hatcheries is given in Table 2. The results showed that the isolation rate of *Salmonella* was more than 40% in seven hatcheries; all of them were San huang breeder chicken hatcheries and Ma breeder chicken hatcheries. *Salmonella* was not detected in hatcheries of imported breeds, including two Cobb hatcheries, five Hyline hatcheries, one Arbor Acres hatchery, and one Ross 308 hatchery. The statistical analysis showed that the prevalence of *Salmonella* in domestic breeds was much higher than that of foreign breeds (*p* < 0.0001). The breed San huang was the most contaminated one (48.60%, *n* = 173, *N* = 356), followed by the breed Ma (34.60%, *n* = 282, *N* = 815), while the breed Hyline present the lowest contamination rate (0.33%, *n* = 1, *N* = 302). However, among the four imported breeds, Ross 308 and Arbor Acres were more susceptible to *Salmonella* contamination than Hyline and Cobb (*p* < 0.0001).

**Table 2:** The distribution of six breeds at different hatcheries.

**Hatcheries**	**Breeds**		**Positive samples**	**%Prev. (BL)^1^**	**%Prev. (Ov.)**
Scale unknown	Ross 308		0	0.00	0.00
S-hatcheries	Hyline		0	0.00	0.00
M-hatcheries	Hyline^****^^a^^,^^b^	**Foreign**	1	0.33	0.05
	Cobb		1	0.47	0.05
	Arbor acres		6	6.12	0.28
	Ross 308		41	11.48	1.92
	San huang	**Domestic**	173	48.60	8.09
	Ma		262	34.60	13.18
L-hatcheries	Hyline		0		
	Ma		20		

1 *Prevalence at breed level*;

**** *p < 0.0001*,

a *statistical difference in Salmonella prevalence between foreign and domestic breeds*;

b *statistical difference in Salmonella prevalence between Arbor Acres, Ross 308 and Hyline, Cobb*.

**Figure 2:**
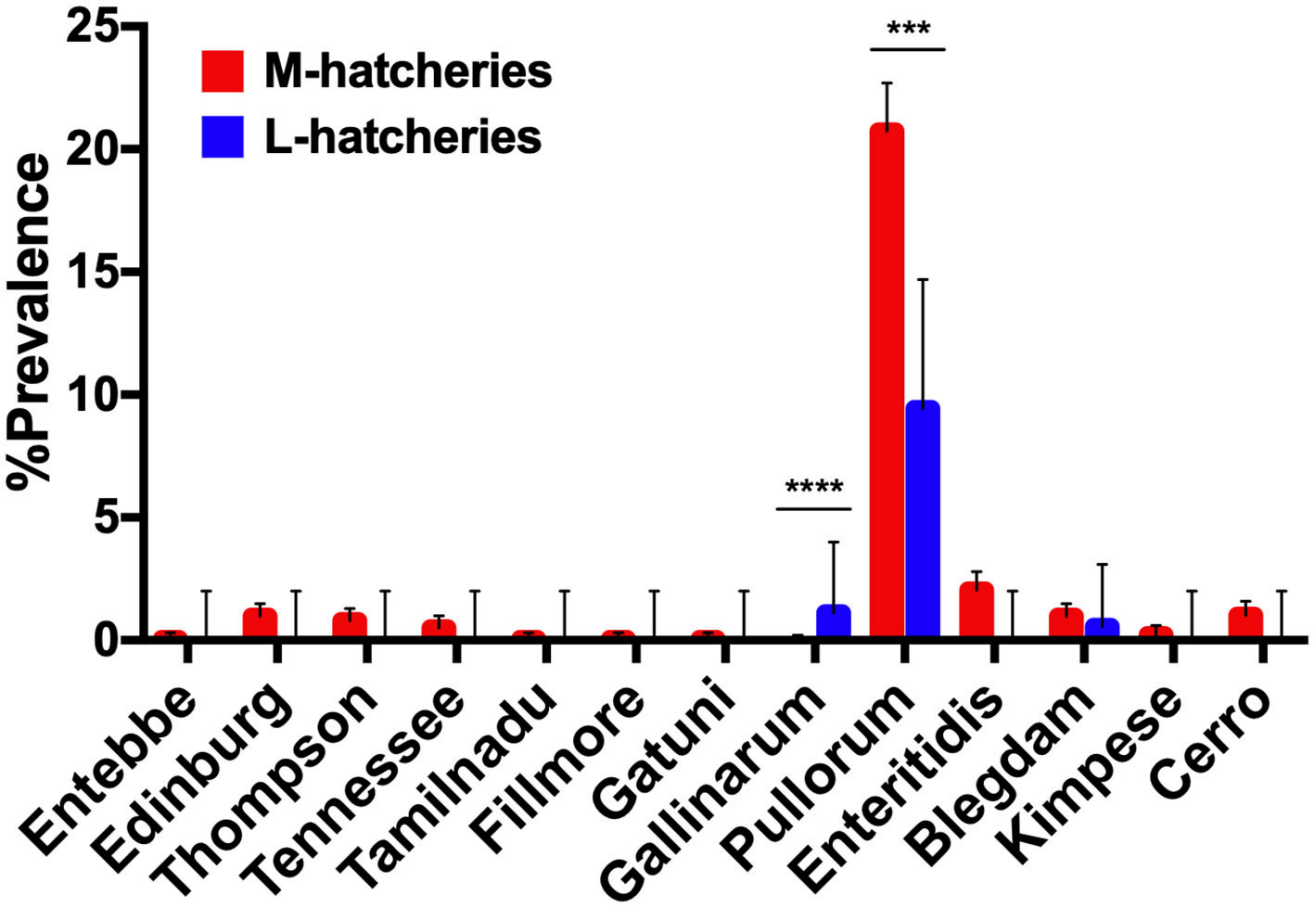
The hatchery scale variation in the distribution of prevalence of 12 serovars. Serogroup: O:4(B), Entebbe; O:7(C1), Edinburg/Thompson/Tennessee/Tamilnadu; O:8(C2-C3), Fillmore/Gatuni; O:9(D1), Gallinarum (Pullorum)/Enteritidis/Blegdam/Kimpese; O:18(K), Cerro. ***The differencee in prevalence rate of *S*. Pullorum between M-hatcheries and L-hatcheries was statistically significant (*p* < 0.001). ****The differencee in prevalence rate of *S*. Gallinarum between M-hatcheries and L-hatcheries was statistically highly significant (*p* < 0.0001).

Among 504 *Salmonella* isolates, 12 different serovars and two biovars of serovar Gallinarum were identified (Table 1). Serovars distribution showed the dominance of *S*. Pullorum (17.86%, *n* = 382), followed by *S*. Enteritidis (1.68%, *n* = 36), while the lowest prevalent serovars were Entebbe, Tamilnadu, Fillmore and Gatuni (0.05%, *n* = 1). *S*. Gallinarum was detected only in L-hatcheries, while many serovars like Entebbe, Edinburg, Thompson, Tennessee, Tamilnadu, Fillmore, Gatuni, Enteritidis, Kimpese, and Cerro were isolated only from M-hatcheries. *S*. Pullorum was isolated at the highest frequency both in M-hatcheries (20.75%, *n* = 365) and L-hatcheries (9.44%, *n* = 17), while *S*. Blegdam was more prevalent (0.97%, *n* = 17) in M-hatcheries. The prevalence of *Salmonella* serovars according to the hatchery scale variation was presented in Figure 2 and revealed that the difference in prevalence rate between M-hatcheries and L-hatcheries was due to *Salmonella* Gallinarum biovars Pullorum (*p* < 0.001) and Gallinarum (*p* < 0.0001).

### Antimicrobial Resistance and Multidrug Resistance Pattern

**Figure 3:**
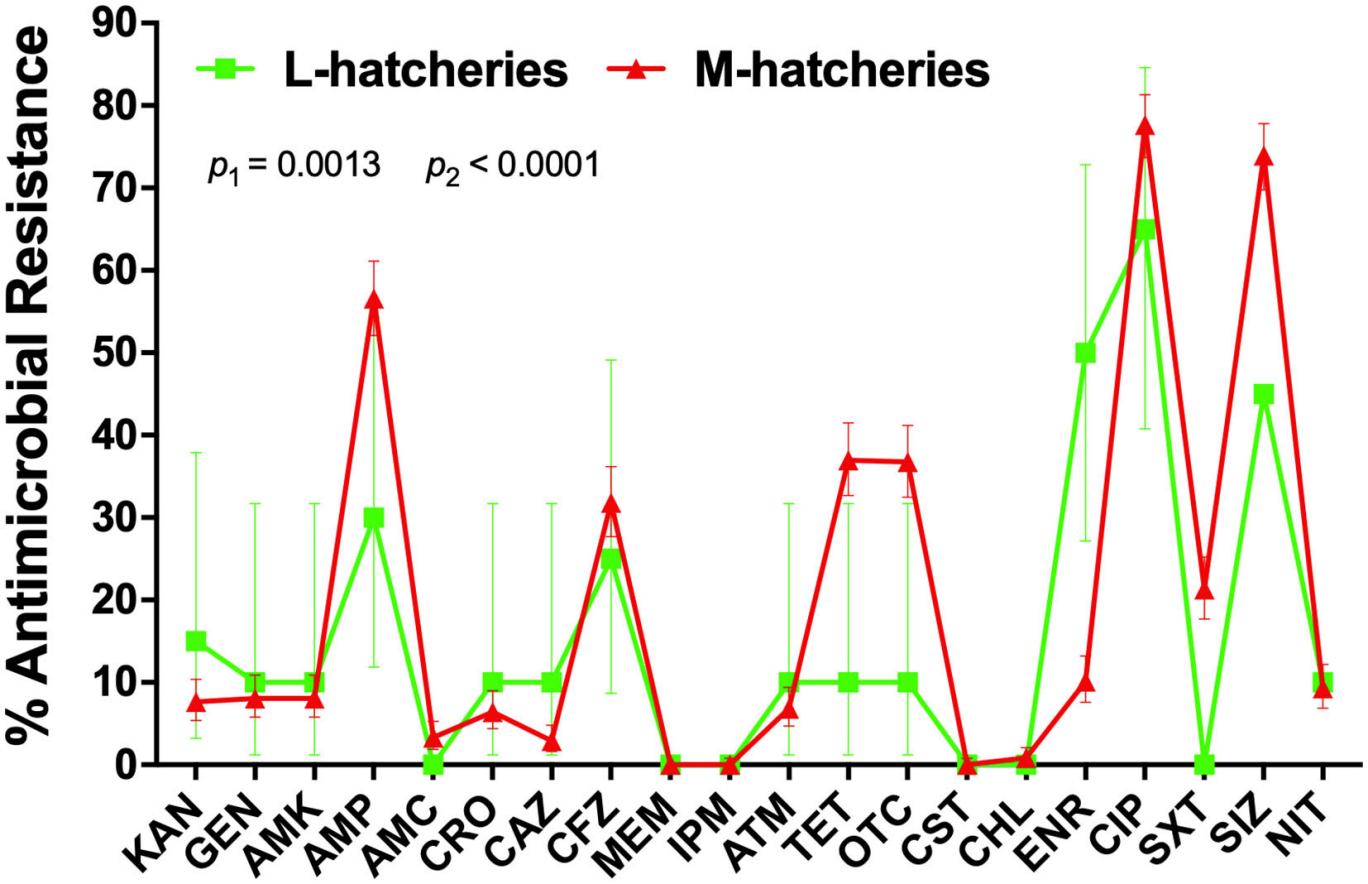
Distribution of the average antimicrobial resistance (in percent) of individual antimicrobials in *Salmonella* isolates from samples collected across L-hatcheries, M-hatcheries. S-hatcheries have no positive samples. The overall average resistance of *Salmonella* isolated from M-hatcheries was higher than that of L-hatcheries (*p* = 0.0013), and the difference in resistance to the individual antimicrobial was statistically highly significant (*p* < 0.0001).

AR patterns of the isolated *Salmonella* strains were determined by Kirby-Bauer (K-B) disc diffusion assay for 20 antimicrobial agents, representing 12 different classes, and the results were presented in Table 3. These results showed that *Salmonella* isolates had high resistance against quinolones (ciprofloxacin, 77.00%) and sulphonamides (sulfisoxazole, 73.00%), but low resistance against phenicols (chloramphenicol, 1.00%), while they were susceptible to carbapenems (meropenem and imipenem) and polypeptides (colistin). Moreover, our results showed that the overall average resistance of *Salmonella* isolated from M-hatcheries was higher than that of L-hatcheries (*p* = 0.0013), while statistical analysis performed by using two-way ordinary ANOVA, showed that the difference in resistance to the individual antimicrobial was statistically highly significant (*p* < 0.0001) (Figure 3).

**Table 3:** Prevalence of Antimicrobial Resistance among *Salmonella* isolates.

**Classes**	**Antimicrobials**	**concentrations (μg)**	***n***	**%Resistance**
Aminoglycosides	KAN	30	40	7.94
	GEN	10	41	8.13
	AMK	30	41	8.13
Penicillin	AMP	10	280	55.56
β-lactams	AMC	20/10	16	3.17
Cephems	CRO	30	33	6.55
	CAZ	30	16	3.17
	CFZ	30	159	31.55
Carbapenems	MEM	10	0	0.00
	IPM	10	0	0.00
Monobactams	ATM	30	35	6.94
Tetracyclines	TET	30	181	35.91
	OTC	30	180	35.71
Polypeptide	CST	10	0	0.00
Phenicol	CHL	30	4	0.79
Quinolones	ENR	5	59	11.71
	CIP	5	389	77.18
Sulphonamides	SXT	23.75/1.25	103	20.44
	SIZ	250	367	72.82
Nitrofurans	NIT	300	47	9.33

**Figure 4:**
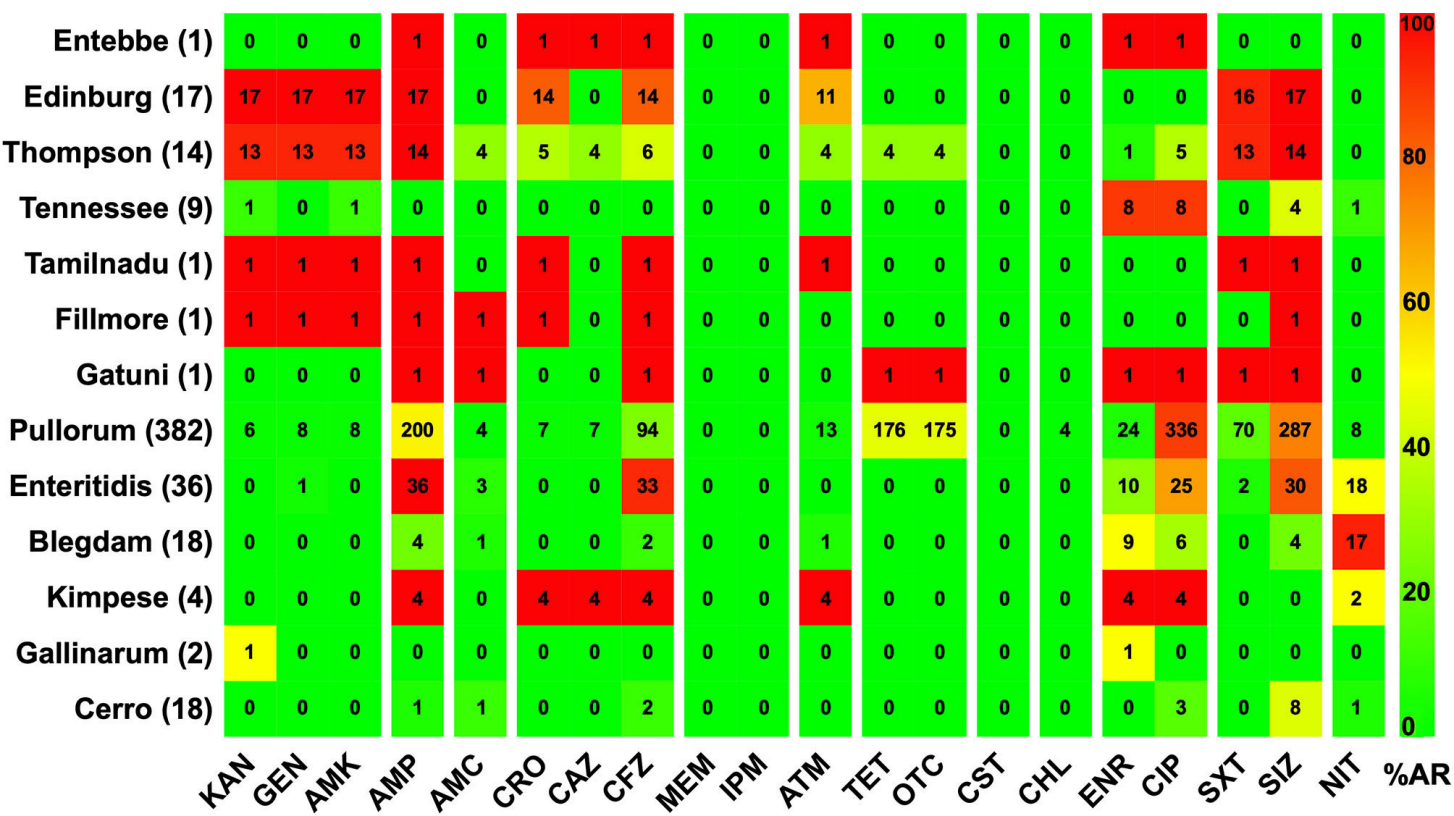
The prevalence of resistance among *Salmonella* isolates against the different classes of antimicrobials. The distribution of the average antimicrobial resistance (in percent) of various serotypes to 20 antimicrobials of 12 different classes independent of the source of the strains. The color of individual cells varies with the percentage of antimicrobial resistance. The number in individual cells represents number of resistant strains (*n*). The names of the antimicrobials (XX′) are abbreviated as KAN, kanamycin; GEN, gentamicin; AMK, amikacin; AMP, ampicillin; AMC, amoxicillin-clavulanic; CRO, ceftriaxone; CAZ, ceftazidime; CFZ, cefazolin; MEM, meropenem; IPM, imipenem; ATM, aztreonam; TET, tetracycline; OTC, oxytetracycline; CST, colistin; CHL, chloramphenicol; ENR, enrofloxacin; CIP, ciprofloxacin; SXT, sulfamethoxazole-trimethoprim; SIZ, sulfisoxazole; NIT, nitrofurantoin.

**Figure 5:**
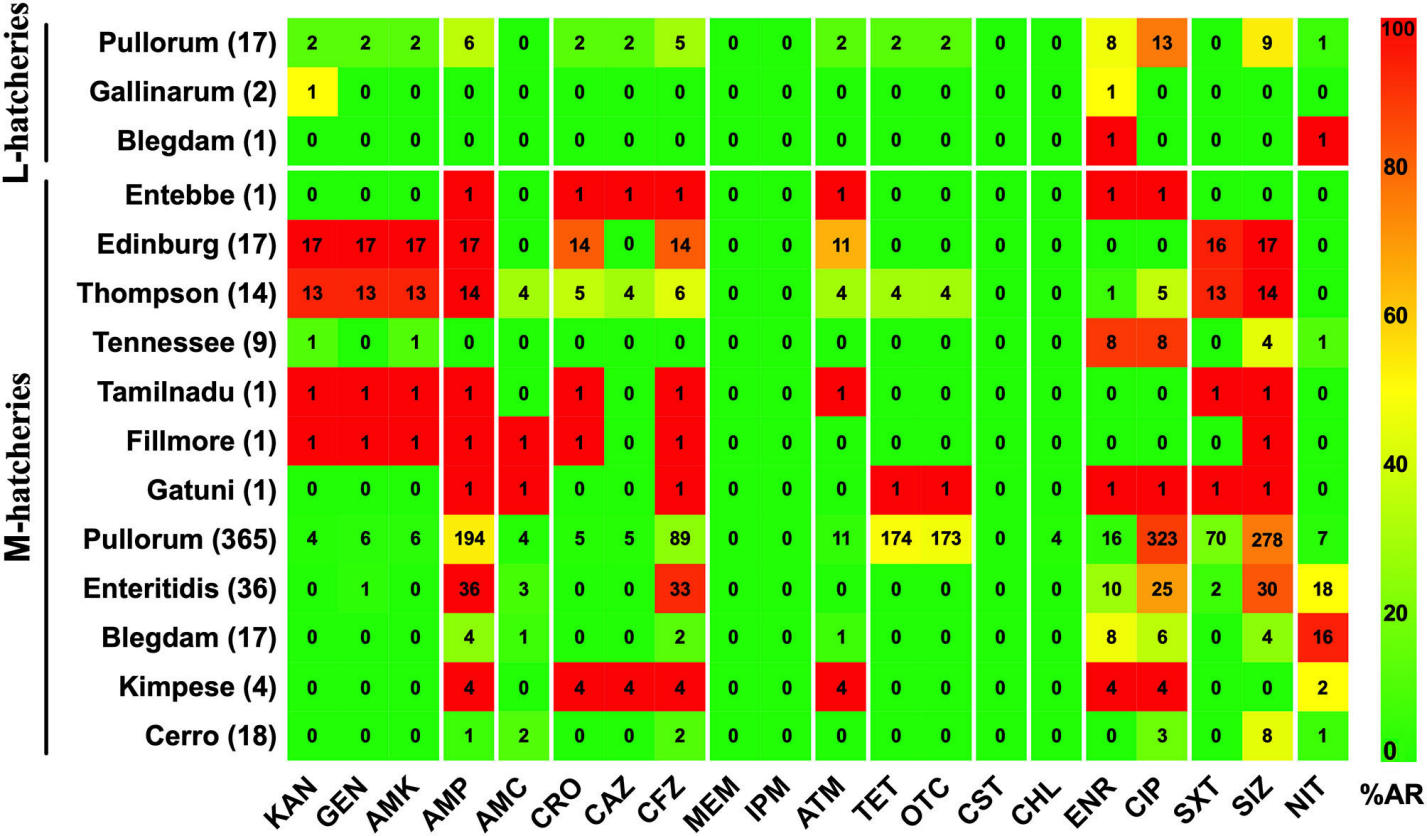
Prevalence of Antimicrobial Resistance and Serovars between L-hatcheries and M-hatcheries. The upper 3 rows are the distribution in the large-scale hatcheries while the lower rows are the distribution in medium-scale hatcheries. The color of individual cells varies with the percentage of antimicrobial resistance. The number in individual cells represents number of resistant strains (*n*).

The AR patterns related to different serovars or biovars are given in Figure 4. These results showed that the variation in the resistance among serovars was statistically significant (*p* < 0.05). It should be noted that only one strain of serovar Entebbe, Tamilnadu, Fillmore, and Gatuni was isolated in this study. Moreover, the resistance of the same serotype was statistically significant when it was compared between L-hatcheries and M-hatcheries (*p* < 0.01) (Figure 5).

**Figure 6:**
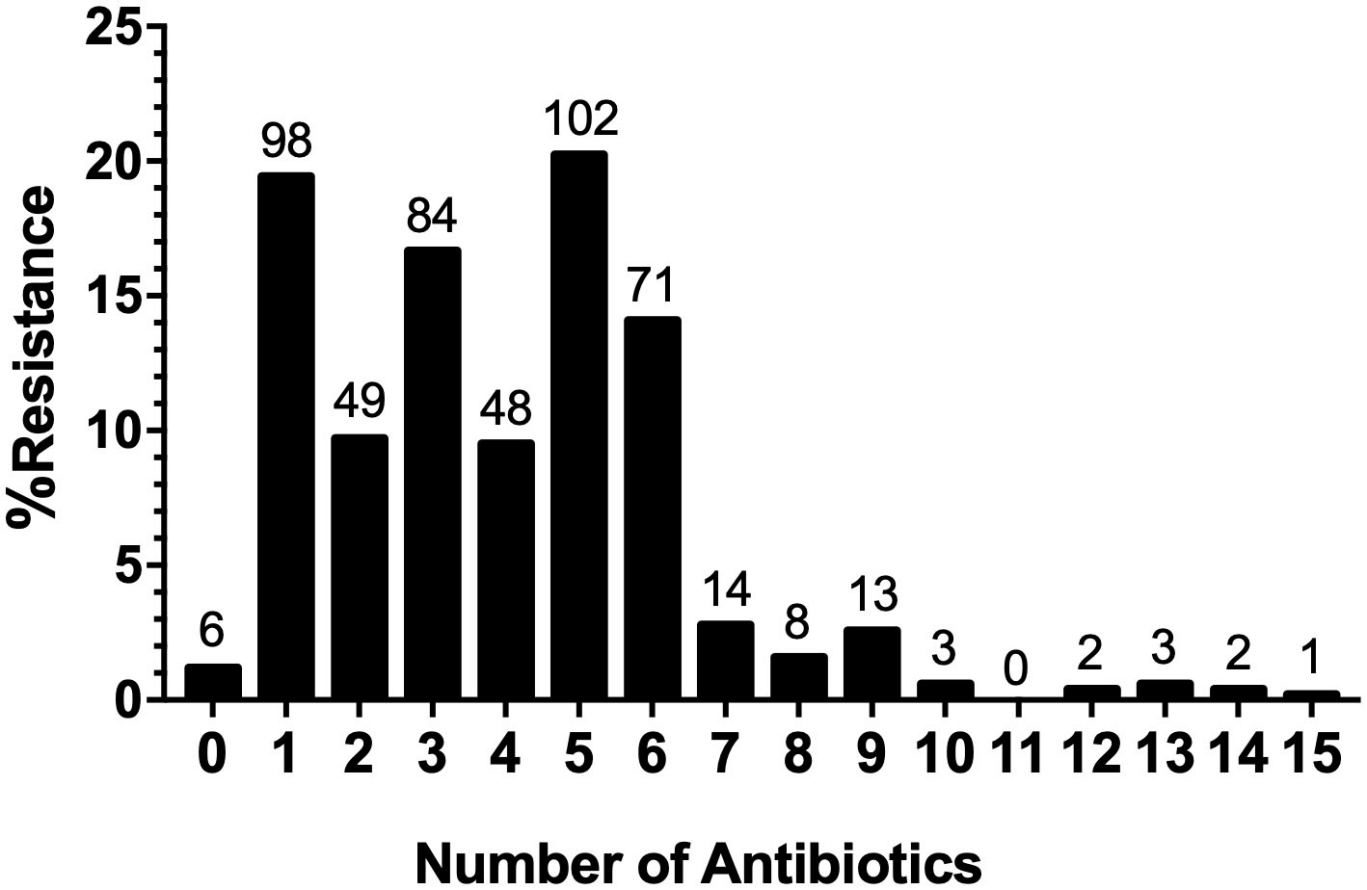
Distribution of Multi-drug resistance (MDR) Strains. MDR patterns for the number of antimicrobials and the distribution of multidrug-resistant strains.

The AR analysis showed that 98.81% (*n* = 498) of the isolates were resistant to at least one antimicrobial and 69.64% (*n* = 351) were resistant to three or more antimicrobial agents. Moreover, we found that the penta-drug resistance pattern was the most represented (20.24%), while the most extensive resistance pattern was observed in one strain of *Salmonella* serovar Thompson (0.20%), which was resistant to 15 antimicrobials belonging to eight different classes (Figure 6).

**Figure 7:**
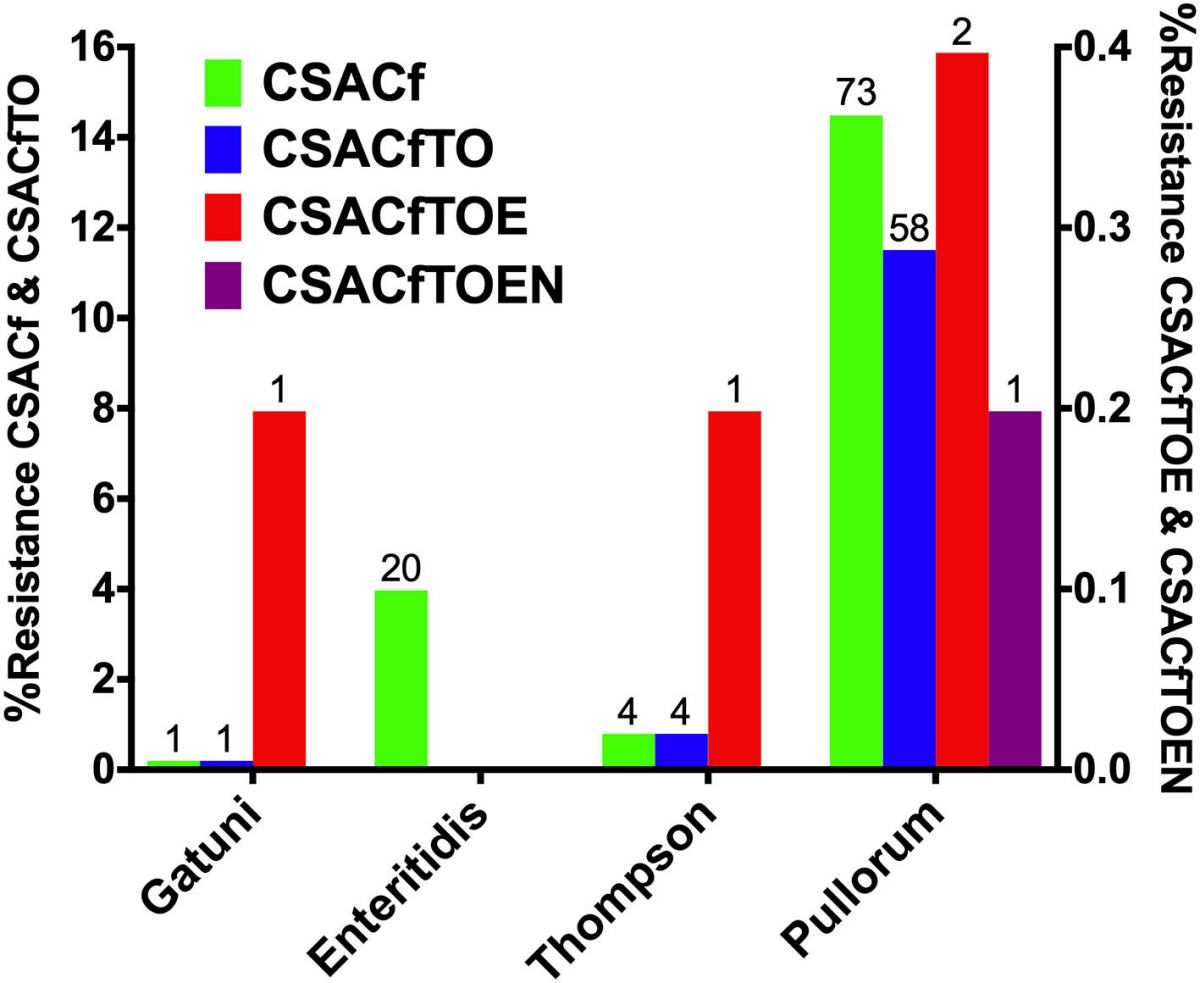
Serovar distribution of Multi-drug resistance (MDR) Prevalence. The left Y-axis shows tetra- and hexa-drug resistance while the right Y-axis shows hepta-drug resistance, except in serovar Enteritidis and octa-drug resistance (see only in *S*. Pullorum).

MDR analysis showed the detection of tetra-, hexa-, hepta-, and octa-drug resistance patterns, which were presented in Figure 7. These MDR patterns were distributed in serovars or biovars Pullorum (382), Thompson (14), Enteritidis (36), and Gatuni (1) which were all recovered from the south of the Yellow River. Tetra-resistance pattern (CSACf, i.e., resistance to ciprofloxacin, sulfisoxazole, ampicillin, and cefazolin) was the most frequently observed (maximum of 14.48% for Pullorum) among the recorded serovars or biovars. Hexa-drug resistance (CSACfTO, i.e., CSACf with tetracycline and oxytetracycline) and hepta-drug resistance (CSACfTOE, i.e., CSACfTO with enrofloxacin) were both highest (11.51% and 0.40%) for *S*. Pullorum. Octa-drug resistance (CSACfTOEN, i.e., CSACfTOE with nitrofurantoin) was recorded only in *S*. Pullorum (0.20%).

**Figure 8:**
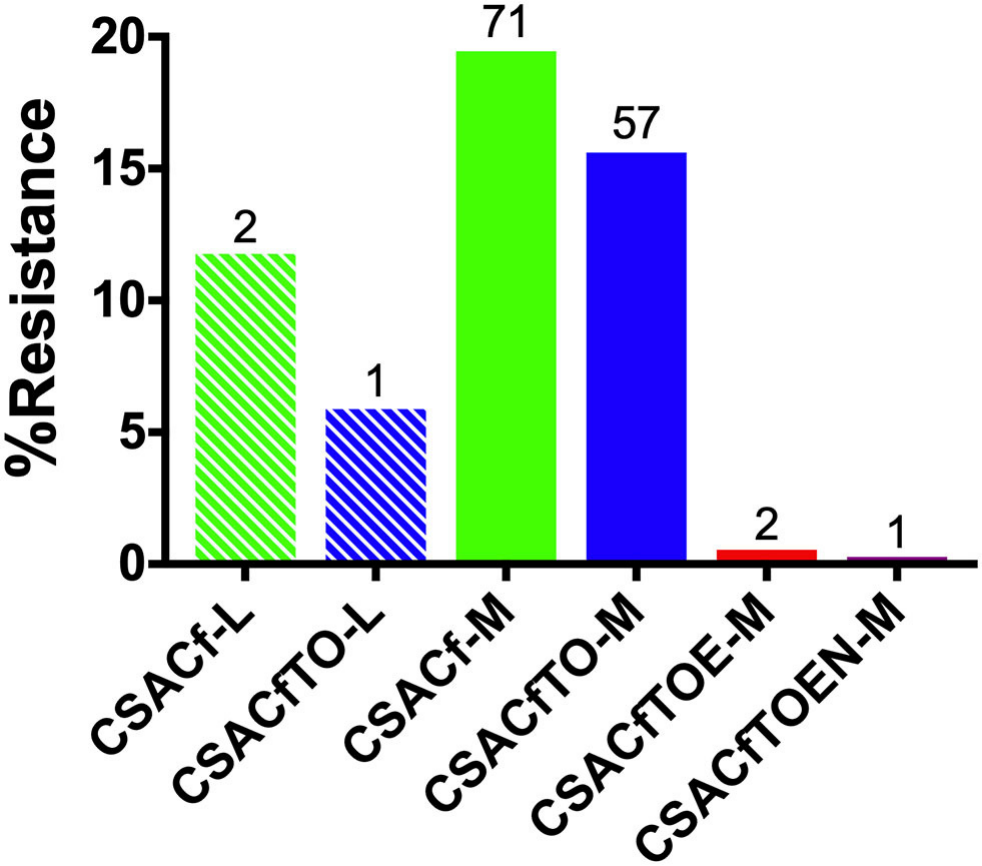
Hatchery scale distribution of multi-drug resistance (MDR) prevalence of Pullorum. Hatchery scale are denoted as medium-scale hatcheries (M) and large-scale hatcheries (L). Due to the low diversity of serotypes isolated from L-hatcheries, there were no Thompson, Enteritidis, or Gatuni isolated from L-hatcheries, so here we only compared the MDR strains of Pullorum in L-hatcheries and M-hatcheries (S-hatcheries had no positive samples).

The distribution of MDR among the isolates was analyzed and compared related to the scale of hatcheries. This analysis was limited only to the *Salmonella* Gallinarum biovar Pullorum, due to the low diversity of serovars isolated from L-hatcheries, and serovars Thompson, Enteritidis and Gatuni were not detected in L-hatcheries. The results showed that the MDR isolates of *S*. Pullorum isolated from M-hatcheries were higher than those reported in L-hatcheries (*p* < 0.0001) (Figure 8).

## Discussion

*Salmonella* is a common pathogen in poultry farms worldwide, which can be transmitted by horizontal and vertical ways causing huge economic losses to the poultry industries every year. In fact, avian salmonellosis can be transmitted vertically through fertilized eggs and causes the death of hatching chicken embryos. In this study, we selected dead embryos instead of the normal embryo samples in order to reflect the actual disease burden of *Salmonella* in breeders.

In this study, the prevalence of *Salmonella* in dead embryos of breeder chickens in Henan province was around 23.56%. These results were similar to those reported in chicken farms in Shandong province, China (24.0%) (Zhao et al., 2017), higher than those reported in El-Menofia province in Egypt (14.3%) (Abdeen et al., 2018), and lower than those reported in the Thailand-Cambodia border provinces (35.75%) (Trongjit et al., 2017). This comparison should be made with suspicion because the prevalence of *Salmonella* may be influenced by several factors like the difference in economic development between countries, samples types, sampling locations, sampling seasons, hygienic quality of the production units, and isolation methods (Kuang et al., 2015; Ed-Dra et al., 2017). Moreover, our results showed that the prevalence of *Salmonella* varies depending on the hatcheries scale. In fact, the isolation rate of *Salmonella* from M-hatcheries was much higher than that of L-hatcheries (*p* < 0.0001), while *Salmonella* isolates were not detected in both S-hatcheries and the unknown-scale hatchery. These results can be explained by the implementation of biosecurity measures in the larger hatcheries which participate in minimizing the prevalence of pathogens. However, *Salmonella* was not detected in S-hatcheries, probably because the scale was very small, which might be conducive to the well-established management and control.

Serovars distribution was performed according to Kauffmann-White scheme and differentiated 12 different serotypes among the 504 *Salmonella* isolates. Two common biovars within serovar Gallinarum were identified. *Salmonella* Gallinarum biovar Pullorum was the most represented (75.79%), followed by Enteritidis (7.14%), Blegdam (3.57%), and Cerro (3.57%). These results demonstrated that there is serious *S*. Pullorum contamination in breeding chicken farms in Henan Province, which were consistent with the earlier studies about *Salmonella* serotypes of avian origin in 12 Chinese provinces (Gong et al., 2014). However, other studies have reported the dominance of other *Salmonella* serovars; *S*. Weltevreden and *S*. Agona in broiler farms (cloacal swab samples) in Guangdong Province (Ren et al., 2016), *S*. Enteritidis (rectal swab samples, chicken embryo samples) in Shandong Province (Zhao et al., 2017), *S*. Derby and *S*. Typhimurium (cloaca swab sampling) in Sichuan Province (Ma et al., 2017). Moreover, *S*. Corvallis and *S*. Typhimurium were described in Thailand (Trongjit et al., 2017), *S*. Typhimurium and *S*. Enteritidis in Egypt and America (Medalla et al., 2016; Mahmoud et al., 2018), *S*. Havana and *S*. Enteritidis in Portugal (Clemente et al., 2014). Therefore, the predominant serotypes of *Salmonella* may vary with time and region of sampling (Kuang et al., 2015). Importantly, the major serovar or biovar isolated in this study (Pullorum) has a major veterinary concern because it is the causative agent of Pullorum disease in chicken, resulting in considerable economic losses to the poultry industries (Geng et al., 2014). However, *S*. Enteritidis have been reported as responsible for many foodborne outbreaks in Henan province, China (Xia et al., 2009). Indeed, *Salmonella* can be transmitted from farms to humans through food products, including meat and eggs (Zhao et al., 2017). Therefore, it is necessary to continuously monitor the local serovar variation of *Salmonella* and formulate a reasonable prevention and control strategy accordingly.

In order to project the prevalence and the distribution of *Salmonella* among the breeder breeds, a total of six breeds were subjected to this study, including four imported breeds, namely Ross 308 (UK), Cobb (USA), Arbor Acres (USA) which are mainly used for meat production and Hyline (USA) mainly used for eggs production, and two local breeds, namely Ma and San huang which are both used for meat and eggs production. Our results showed that the prevalence of *Salmonella* in domestic breeds was much higher than that of foreign breeds (*p* < 0.0001). Moreover, the serovars distribution among domestic breeds were consistent with the dominance of *S*. Pullorum, while only one strain of *S*. Pullorum was detected in the imported breed. To a certain extent, it was indicated that *S*. Pullorum has been decontaminated thoroughly of foreign breed chicken (Lu et al., 2014), while the Chinese poultry industries still suffered from serious *S*. Pullorum contamination (Gong et al., 2014). Among these various breeds, the prevalence of *Salmonella* in San huang and Ma was the highest (48.60 and 34.60%, respectively) with additional serovars or biovar distribution (*n* = 11) in Ma chicken breed. However, only one *Salmonella* strain was isolated from Hyline and Cobb, respectively, while the prevalence of *Salmonella* in Ross 308 was more serious (11.48%) than the other imported breeds. Notably, we found that 34 of the 41 *Salmonella* isolates from Ross 308 were recovered from the same hatchery, with an isolation rate of 28.33%. However, the breeding farm corresponding to the hatchery was in a poor sanitary environment, which *S*. Enteritidis has been detected in this farm before entering the chicks. Therefore, the prevalence of *Salmonella* in different chicken farms was related to the chicken breed, feeding management and sanitation environment. The prevalence of *Salmonella* in LH-1, LH-2, and ZZ-6 hatcheries was 5.26, 15.00, and 8.26%, respectively, which is lower than that of other hatcheries of Ma and San huang breeds. After investigation, we found that varying degrees of *S*. Pullorum quarantine and purification had been carried out in these three hatcheries. It was showed that quarantine and purification had a significant effect on the control of *S*. Pullorum and suggested that the hatcheries and farms should pay particular attention to the introduction of healthy chicks, strict disinfection, and regular quarantine to eliminate positive eggs and chickens, so as to reduce the prevalence of *Salmonella*.

AR prevalence of *Salmonella* to ciprofloxacin (77.00%), sulfisoxazole (73.00%), ampicillin (55.60%), tetracycline (36.00%), and oxytetracycline (36.00%) exceeded the 36%, which was similar to the AR situation of Sichuan, Shandong, Guangdong, and Shanxi provinces (Li et al., 2013; Yang et al., 2014; Zhao et al., 2017). As well as some imported studies were reported the high resistance of *Salmonella* isolates to ampicillin, tetracycline, ciprofloxacin, and sulfonamides (Bacci et al., 2012; Clemente et al., 2014; Abdeen et al., 2018; Chuah et al., 2018), which indicate that *Salmonella* of poultry origin from several countries has developed a high resistance to traditional antimicrobials such as quinolones, penicillins, sulfonamides, and tetracyclines which are widely used in livestock and poultry farming to treat bacterial avian diseases and to promote growth (Mehdi et al., 2018). However, our findings showed that the *Salmonella* isolates were susceptible to meropenem, imipenem, and presented a low resistance to chloramphenicol, with only one strain of *S*. Pullorum, in fact, the use of these antimicrobials was banned by veterinarians, indicating that the strengthening of veterinary medicine management can effectively avoid the development of AR bacteria. Moreover, the low resistance of isolated strains to amoxicillin-clavulanic acid, ceftazidime, aztreonam, and nitrofurantoin may be related to the seldom use of these antimicrobials in animal production.

Different serovars and biovars of *Salmonella* showed different AR patterns. The antimicrobial resistance of serovar Edinburg and Thompson were alarming high, which showed high antimicrobial resistance rates, with a wide antimicrobial resistance spectrum. Although these two are not the dominant serovars, the prevalence of serovars may change over time and they are likely to be the dominant serovars under the multiple antimicrobial selection pressure (Rahmani et al., 2013). Pullorum as the dominant serovar showed very high resistance to quinolones (ciprofloxacin) and sulphonamides (sulfisoxazole), which were commonly used in farms. Similarly, Enteritidis as the second dominant serovar of these isolates, showed high resistance to penicillin (ampicillin), cephems (cefazolin), quinolones (ciprofloxacin), and sulphonamides (sulfisoxazole), which were frequently used in farms. These also supported that most of the *Salmonella* pathogens presented in farms have a common antimicrobial resistance pattern (a Tetra-resistance pattern toward ciprofloxacin, sulfisoxazole, ampicillin, and cefazolin), suggesting that veterinarians in farms need to improve the traditional medication regimen. Tennessee and Blegdam had high resistance to quinolones (enrofloxacin, ciprofloxacin) and nitrofurans (nitrofurantoin), respectively, but were sensitive to other antimicrobials. Compared with serovars discussed above, Cerro as a relatively uncommon serovar, was sensitive to various antimicrobials. Here, we also found a few minority serovars, including Entebbe, Tamilnadu, Fillmore, Gatuni, Kimpese, and Gallinarum biovar Gallinarum.

This study showed a high prevalence of MDR patterns among the isolated *Salmonella* strains. In fact, 351 (69.64%) *Salmonella* isolates present resistance to three or more antimicrobial agents. These results were in accordance with numerous worldwide studies reporting the widespread of MDR among the isolated *Salmonella* strains (Li et al., 2013; Lu et al., 2014; Moawad et al., 2017; Abdeen et al., 2018; Chuah et al., 2018). However, the intensive use of antimicrobials in both veterinary and medical fields has led to a quite high AR by exerting a selection pressure against the used antimicrobials. Face the issue of the spread of AR bacteria, the government of China has established the program “Pilot Programme for Reduction of Veterinary Antimicrobial Use (2018-2021)” in April 2018 mentioning all pharmaceutical feed additives which would be withdrawn in 2020 (MOA, 2018). Therefore, the use of pharmaceutical feed additives should be gradually reduced and stopped in clinical practice, and the administration of alternatives medication should be strengthened. Furthermore, the use of veterinary banned drugs should be strictly prohibited, and the rotation of medication, alternating medication, and combination of Chinese and Western medicines should be implemented to avoid severe drug resistance caused by prolonged use of the same drugs.

## Conclusion

This study expands the knowledge of epidemiology and AR prevalence of *Salmonella* strains recovered from breeder chicken hatcheries in Henan province, China. Our findings indicate serious contamination of samples by *Salmonella* especially for domestic breeds from M-hatcheries. The dominance of *S*. Pullorum and *S*. Enteritidis is with a major concern for veterinary and food safety fields. Additionally, the detection of MDR *Salmonella* (tetra-, hexa-, hepta-, and octa-drug resistance patterns) is alarming, which requires the implementation of an antimicrobial management plan for rational uses of critical antimicrobials in chicken farms.

## Data Availability Statement

The original contributions presented in the study are included in the article/supplementary material, further inquiries can be directed to the corresponding author/s.

## Author Contributions

YX and ZJ collected the samples. YX, ZJ, and YQ did the lab analysis. YX and XZ did the data analysis and prepared a draft. AE made the comments and corrections of the draft. MY conceived the project and provided critical comments for the draft. All authors have read and agreed to the manuscript.

## Conflict of Interest

The authors declare that the research was conducted in the absence of any commercial or financial relationships that could be construed as a potential conflict of interest.
